# Baseline perihematomal edema, C-reactive protein, and 30-day mortality are not associated in intracerebral hemorrhage

**DOI:** 10.3389/fneur.2024.1359760

**Published:** 2024-04-05

**Authors:** Oluwaseun A. Sobowale, Isabel C. Hostettler, Teddy Y. Wu, Calvin Heal, Duncan Wilson, Darshan G. Shah, Daniel Strbian, Jukka Putaala, Turgut Tatlisumak, Andy Vail, Gagan Sharma, Stephen M. Davis, David J. Werring, Atte Meretoja, Stuart M. Allan, Adrian R. Parry-Jones

**Affiliations:** ^1^Division of Cardiovascular Sciences, School of Medical Sciences, Manchester Academic Health Science Center, University of Manchester, Manchester, United Kingdom; ^2^Geoffrey Jefferson Brain Research Center, Manchester Academic Health Science Center, Northern Care Alliance NHS Foundation Trust and University of Manchester, Manchester, United Kingdom; ^3^Stroke Research Center, UCL Queen Square Institute of Neurology, and the National Hospital for Neurology and Neurosurgery, University College London Hospitals NHS Foundation Trust, London, United Kingdom; ^4^Department of Neurosurgery, Cantonal Hospital St. Gallen, St. Gallen, Switzerland; ^5^Department of Medicine and Neurology, The Royal Melbourne Hospital, University of Melbourne, Parkville, VIC, Australia; ^6^New Zealand Brain Research Institute, Christchurch, New Zealand; ^7^Center for Biostatistics, The University of Manchester, Manchester Academic Health Science Center, Manchester, United Kingdom; ^8^Department of Medicine, Princess Alexandra Hospital, Brisbane, QLD, Australia; ^9^Department of Neurology, Helsinki University Hospital and Helsinki University, Helsinki, Finland; ^10^Department of Clinical Neuroscience, Institute of Neuroscience and Physiology, Sahlgrenska Academy at University of Gothenburg, Gothenburg, Sweden; ^11^Department of Neurology, Sahlgrenska University Hospital, Gothenburg, Sweden; ^12^Division of Neuroscience, School of Biological Sciences, Faculty of Biology, Medicine and Health, The University of Manchester, Manchester, United Kingdom

**Keywords:** intracerebral hemorrhage, perihematomal edema, inflammation, C-reactive protein, mortality

## Abstract

**Background:**

The relationship between baseline perihematomal edema (PHE) and inflammation, and their impact on survival after intracerebral hemorrhage (ICH) are not well understood.

**Objective:**

Assess the association between baseline PHE, baseline C-reactive protein (CRP), and early death after ICH.

**Methods:**

Analysis of pooled data from multicenter ICH registries. We included patients presenting within 24 h of symptom onset, using multifactorial linear regression model to assess the association between CRP and edema extension distance (EED), and a multifactorial Cox regression model to assess the association between CRP, PHE volume and 30-day mortality.

**Results:**

We included 1,034 patients. Median age was 69 (interquartile range [IQR] 59–79), median baseline ICH volume 11.5 (IQR 4.3–28.9) mL, and median baseline CRP 2.5 (IQR 1.5–7.0) mg/L. In the multifactorial analysis [adjusting for cohort, age, sex, log-ICH volume, ICH location, intraventricular hemorrhage (IVH), statin use, glucose, and systolic blood pressure], baseline log-CRP was not associated with baseline EED: for a 50% increase in CRP the difference in expected mean EED was 0.004 cm (95%CI 0.000–0.008, *p* = 0.055). In a further multifactorial analysis, after adjusting for key predictors of mortality, neither a 50% increase in PHE volume nor CRP were associated with higher 30-day mortality (HR 0.97; 95%CI 0.90–1.05, *p* = 0.51 and HR 0.98; 95%CI 0.93–1.03, *p* = 0.41, respectively).

**Conclusion:**

Higher baseline CRP is not associated with higher baseline edema, which is also not associated with mortality. Edema at baseline might be driven by different pathophysiological processes with different effects on outcome.

## 1 Introduction

Intracerebral hemorrhage (ICH) is a devastating condition with limited treatment options (1). Primary neurological injury results from direct physical tissue damage caused by the formation of the hematoma and subsequent expansion (2, 3). Secondary injury results from a cascade of cellular and molecular processes, initiated by both brain tissue injury and the presence of blood in the brain parenchyma. Secondary injury contributes to perihematomal edema (PHE) formation and mass effect. The inflammatory response is a major component of this and the modulation of inflammation may represent an important therapeutic strategy in ICH (4).

Computed tomography (CT) of the brain in patients with ICH at baseline typically shows low Hounsfield units (HU) in the perihematomal brain, also seen as high signal on T_2_-weighted magnetic resonance (MR) imaging, which is thought to be caused by PHE. Recent studies using large ICH cohorts have found growth of PHE in the first 24–72 h to be associated with poor outcome (5–7) but few studies have focused on PHE at baseline (<24 h from onset). Baseline PHE is likely to be driven by hydrostatic pressure associated with an expanding hematoma and the coagulation of extravasated whole blood, with the production of coagulum and serum accumulating in the perihematomal brain parenchyma (8). Subsequent growth (beyond 24 h) is thought to be mediated by vasogenic edema resulting from inflammation induced blood–brain barrier disruption (9). Features of systemic inflammation such as elevated C-reactive Protein (CRP) (10–13), fever (14, 15), and peripheral leukocytosis (16, 17) are often observed in ICH patients and are associated with worse clinical outcomes. CRP is an acute phase reactant induced by interleukin-6 (10), which reflects both systemic and local inflammation (18). However, given different proposed mechanisms of baseline PHE, it remains unclear what role systemic inflammation plays in baseline PHE, as the systemic inflammatory response to ICH takes hours to evolve (18). Baseline systemic inflammation is likely to be more reflective of pre-morbid health and may influence outcome from ICH through different mechanisms.

Our aim was to determine the associations between baseline CRP, as a surrogate marker for inflammation, and baseline EED, first in a unifactorial analysis in the variables true form and then after transforming key variables and adjusting for appropriate confounding variables. In a further step, we tested for an independent association of these measures with 30-day mortality.

## 2 Methods

### 2.1 Patients

We conducted a retrospective analysis of a combined cohort of patients from four ICH registries from Salford Royal NHS Foundation Trust (SRFT, United Kingdom), Helsinki University Hospital (Helsinki, Finland), Royal Melbourne Hospital (RMH, Melbourne, Australia) and the Clinical Relevance of Microbleeds in Stroke (CROMIS-2) study. The SRFT registry is a prospective registry of consecutive ICH patients admitted from January 2013 to June 2019. The Helsinki ICH study is a retrospective analysis of consecutive patients treated at Helsinki University Hospital between January 2005 and March 2010 (19). The RMH ICH study included consecutive patients admitted between October 2007 and January 2012 (20, 21). The CROMIS-2 study was a prospective, multicenter observational study that recruited spontaneous ICH patients between 2011 and 2015 from 78 centers in the United Kingdom and one center in the Netherlands (22).

We included patients with baseline CRP measured in the clinical laboratory at the admitting hospital and a baseline CT scan performed within 24 h of symptom onset or last known well time in the present analysis. Follow-up CT scans were not evaluated as these were only available for a small subset of patients. For CROMIS-2 patients, only the date and not the time of ICH onset, CT scans, and CRP samples were recorded. Patients were only included in the analysis if all three dates were the same, to ensure that all CT scans and CRP measurements were within 24 h of onset. We excluded patients with ICH associated with anticoagulant use or systemic thrombolysis because baseline edema may be dependent on the functioning of the clotting cascade and inclusion of such cases would confound interpretation of our analyses. ICH secondary to vascular malformations, trauma, underlying tumor, hemorrhagic transformation of ischemic stroke, primary subarachnoid hemorrhage from any cause including aneurysmal rupture were also excluded.

### 2.2 Clinical data

Patient demographic and clinical data were collected as part of routine care in each clinical registry. Pre-specified data variables were requested from each participating registry which were demographic, clinical [time of symptom onset, baseline National Institutes of Health Stroke Scale (NIHSS), and baseline Glasgow Coma Scale (GCS)], radiological (hematoma location, presence of intraventricular hemorrhage, baseline PHE, and hematoma volume), laboratory (baseline glucose, baseline CRP), medication use at time of stroke (statins, anticoagulants), and survival status at 30 days from symptom onset. Each registry provided anonymized data to the data coordinating and processing center at SRFT.

### 2.3 Hematoma and edema planimetric volume ascertainment

Hematoma and edema volumes were segmented at each center using semi-automated planimetry using previously published methods using either Analyze (Biomedical Imaging Resource; Mayo Clinic) or Osirix (Pixmeo, Geneva, Switzerland) (23, 24). Briefly, de-identified CT images were loaded on to planimetric software in the Digital Imaging and Communications in Medicine format. Edema volume was segmented using a fixed lower HU of 5 and a flexible upper limit with a ceiling of 33 HU, comparing to the unaffected hemisphere for visual estimate of edema vs. leukoaraiosis. The HU range was kept within 44–100 HU for hematoma segmentation. Three authors performed segmentation blinded to clinical information (O.A.S. for Salford cohort, T.Y.W. for Melbourne and Helsinki cohorts and I.C.H. for CROMIS-2 cohort). Our previous work has demonstrated excellent inter-rater and intra-rater reliability for both edema and ICH segmentation using our approach (24).

### 2.4 Edema extension distance

Edema extension distance (EED) is a recently proposed edema metric (25, 26), which calculates the average thickness in centimeters of the edema surrounding the hematoma. The EED is relatively independent of hematoma volume and is calculated using the following formula:


$$\sqrt[{3}]{\frac{\;Edema\;volume\;+\;ICH\;volume\;}{\frac{4}{3}\;\pi }}-\sqrt[{3}]{\frac{\;ICH\;volume\;}{\frac{4}{3}\;\neq }}$$

We used the EED as the outcome variable in a linear regression model to study factors associated with baseline PHE as the EED is relatively independent of the hematoma volume when compared to absolute PHE volume and thus a better indication of PHE alone (25, 26). We used absolute PHE volume as a co-factor in a Cox-regression model with time to death as the outcome as we expect PHE to lead to death by mass effect and raised ICP, thus absolute PHE volume is preferred over EED when death is the outcome.

### 2.5 Statistical analysis

Standard descriptive statistics were used: we present continuous variables using mean (standard deviation; SD) and categorical variables using frequency and percentages. We initially assessed the unifactorial association between baseline CRP and baseline edema using simple linear regression. To determine the unifactorial association between 30-day mortality and each of baseline PHE, EED and CRP we used a series of Mann–Whitney U tests. We then transformed ICH, PHE, and CRP using the natural logarithm to satisfy statistical normal distribution assumptions. We then performed multifactorial analyses to assess the association between baseline CRP and baseline edema, and the association between baseline edema, CRP and 30-day mortality, adjusting for center in all analyses.

#### 2.5.1 Association between baseline CRP and edema

We performed a multifactorial linear regression model with baseline EED as outcome variable, baseline natural logarithm of CRP as the variable of interest. Covariates, chosen based on previous studies where they have been shown to be associated with measures of edema or where there was deemed to be a biological rationale for a potential association, were (5–7, 25, 26): cohort, age, sex, natural logarithm of ICH volume, ICH location, presence of IVH, baseline systolic blood pressure, pre-stroke statin use, and baseline glucose. When CRP was below the sensitivity of the assay used in the hospital laboratory, we imputed a value halfway between zero and the lower limit of assay sensitivity. To determine whether time from onset to measurement altered the association between the variable of interest and the outcome variable, we conducted a sensitivity analysis adding time to CRP measurement and time to baseline CT to the multifactorial model in a subset of patients where this information was available.

#### 2.5.2 Association between baseline CRP, PHE, and risk of death

A multifactorial Cox regression model was used to examine the association between baseline CRP and mortality up to 30 days as the outcome variable and baseline natural logarithm of PHE volume as the variable of interest. Again, covariates were chosen as, based on previous studies, they are all well-established variables influencing mortality risk after ICH. Covariates used in this model were: cohort, baseline natural logarithm of CRP, GCS, sex, age, natural logarithm of ICH volume, ICH location, presence of IVH, baseline systolic blood pressure, pre-stroke statin use, and baseline glucose as covariates.

All data are expressed as median and interquartile range, unless otherwise stated. We set the level of significance to 5% (*p* = 0.05). All statistical analysis were performed using STATA 15 (StataCorp. 2011. *Stata Statistical Software: Release 15*. College Station, TX: StataCorp LP). We report this study following the Strengthening the Reporting of Observational Studies in Epidemiology (STROBE) guidelines.

## 3 Results

### 3.1 Baseline characteristics

Our initial sample had a total of 3,412 ICH patients. Patients were excluded from the final analysis if they had one or more of the following exclusion criteria: missing outcome (*n* = 605), missing CRP value within 24 h from symptom onset (*n* = 1,335), missing other independent variables (*n* = 1,263), no first scan within 24 h from symptom onset (*n* = 93), and/or being on anticoagulation (*n* = 762). Our final cohort included 1,034 patients (Helsinki *n* = 591/1,013, CROMIS-2 *n* = 273/1,024, SRFT *n* = 91/828, RMH *n* = 79/545). Table 1 summarizes the baseline characteristics. Median age was 69 (IQR 59–79) and 463 patients (45%) were female. The median baseline GCS was 14 (IQR 12–15). The median baseline ICH volume was 11.4 (IQR 4.3–28.7) mL; most hematomas were either deep (55%) or lobar (34%) with a small proportion of hematomas in the posterior fossa (brainstem 5%, cerebellum 6%). Intraventricular extension was present in 379 patients (37%). Thirty-day mortality was 19.5% (202 patients).

**Table 1:** Baseline characteristics of sample used in primary analysis.

	Total
	*n* = 1,034
Age, years	69 (59–79)
Female sex, *N* (%)	463 (45%)
Baseline GCS, med (IQR)	14 (12–15)
Baseline hematoma volume, mL med (IQR)	11.4 (4.3–28.7)
Mean baseline systolic BP, mmHg (SD)	173.2 (32.3)
Baseline glucose, med (IQR)	6.95 (5.9–8.5)
Baseline CRP, med (IQR)	2.5 (1.5–7)
Baseline EED, cm, med (IQR)	0.37 (0.21–0.51)
Statin use, *N* (%)	216 (21%)
Hematoma location, *N* (%)	
Lobar	352 (34%)
Deep	572 (55%)
Brainstem	53 (5%)
Cerebellum	57 (6%)
IVH, *N* (%)	379 (37%)
30-day mortality, *N* (%)	202 (19.5%)

All data expressed as median (interquartile range) or mean as stated.

BP, Blood pressure; CRP, C-reactive protein; cm, Centimeter; EED, Edema extension distance; GCS, Glasgow Coma Scale; IVH, Intraventricular hemorrhage; and mL, Milliliter.

### 3.2 Association between CRP and EED

Median CRP-value within 24 h was 2.5 (IQR 1.5–7.0) mg/L. 422 (40.8%) CRP measurements were below assay sensitivity and were thus imputed as described in the methods section. The median baseline EED was 0.37 cm (IQR 0.21–0.51).

**Figure 1:**
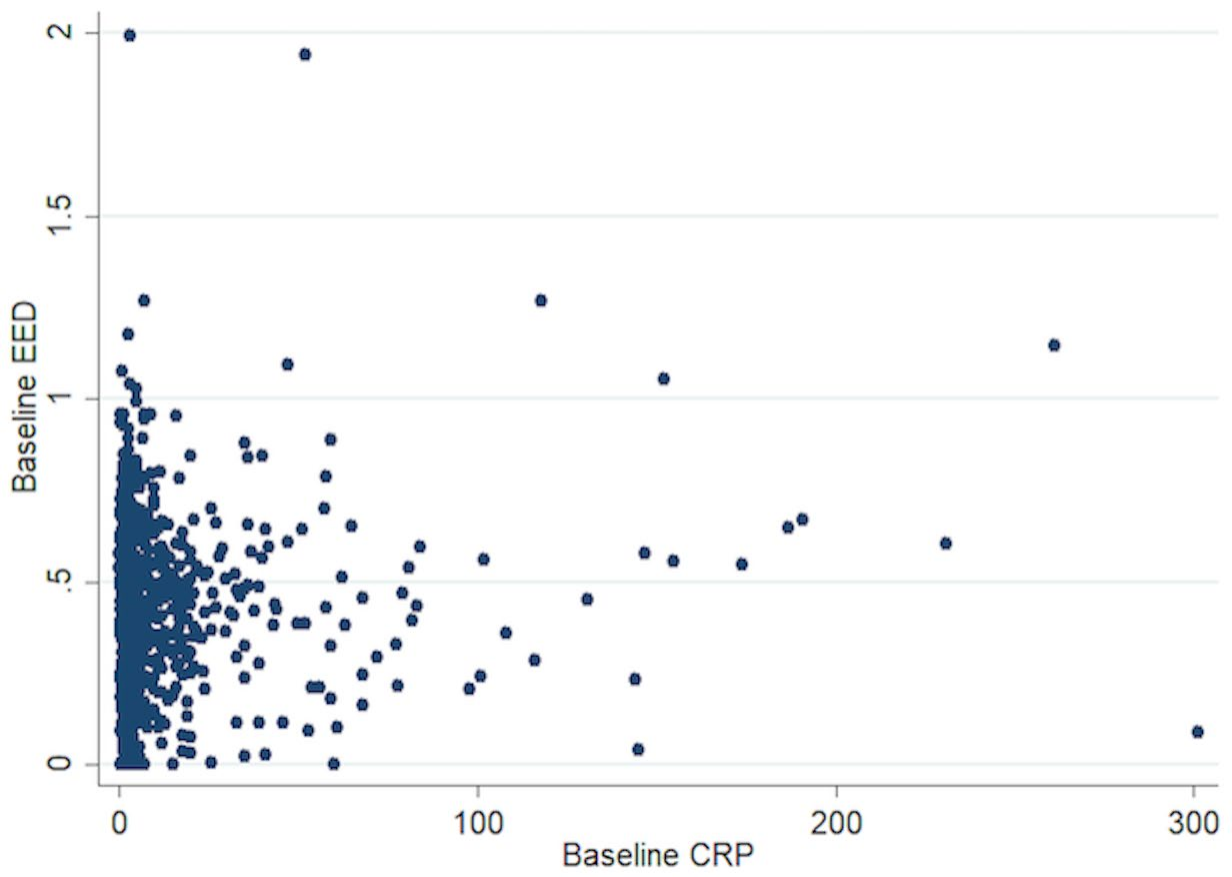
Scatter plot association baseline CRP and EED. CRP, C-reactive protein; EED, Edema extension distance.

In the unifactorial analysis, higher baseline CRP was associated with a higher baseline EED of 0.012 cm per 10 units of CRP (95% CI 0.000–0.018, *p* < 0.001, Figure 1), but once we transformed the variables and adjusted for confounding factors this relationship was no longer significant. In a multifactorial linear regression model, higher baseline log-CRP was not independently associated with increased baseline EED: for a 50% increase in baseline CRP the difference in expected mean baseline EED was 0.004 cm (95%CI— < 0.001–0.008, *p* = 0.055). Increasing age (0.001, 95%CI <0.001–0.002, *p* = 0.02) and baseline ICH volume [for a 50% increase in baseline ICH, baseline EED increased on average 0.009 (95%CI 0.008–0.011), *p* < 0.001] were associated with higher baseline EED while male sex (−0.029, 95%CI −0.052 to −0.006, *p* = 0.02), presence of IVH (−0.067, 95%CI −0.093 to −0.040, *p* < 0.001), brainstem (−0.203, 95%CI −0.260 to −0.145, *p* = 0.01), or cerebellar (−0.124, 95%CI −0.179 to −0.070, *p* < 0.001) location were associated with lower baseline EED (Table 2).

**Table 2:** Multifactorial linear regression model assessing the association between baseline C-reactive protein and EED.

	Coefficient	SE	95% CI	*p*- value
logCRP	0.010	0.005	<−0.001 to 0.027	0.055
Male sex	−0.029	0.015	−0.052 to −0.006	0.015
Age	0.001	0.016	<0.001 to 0.002	0.016
logICH volume	0.052	0.005	0.043–0.062	<0.001
IVH	−0.067	<0.001	−0.093 to −0.040	<0.001
Location				
Lobar	-	-	-	-
Deep	−0.015	0.014	−0.042 to 0.121	0.279
Brainstem	−0.203	0.014	−0.260 to 0.145	<0.001
Cerebellum	−0.124	0.028	−0.179 to −0.070	<0.001
Statin use	−0.010	0.015	−0.039 to 0.018	0.475
Baseline glucose	<0.001	0.001	−0.002 to 0.004	0.599
Baseline systolic BP	<−0.001	<0.001	<−0.001 to <0.001	0.176
Cohort				
Helsinki	ref.	-	-	-
CROMIS	0.205	0.014	0.176–0.232	<0.001
Melbourne	0.097	0.023	0.052–0.143	<0.001
Salford	−0.130	0.021	−0.172 to −0.088	<0.001

BP, Blood pressure; CRP, C-Reactive protein; EED. Edema extension distance; and IVH, Intraventricular hemorrhage.

**Figure 2:**
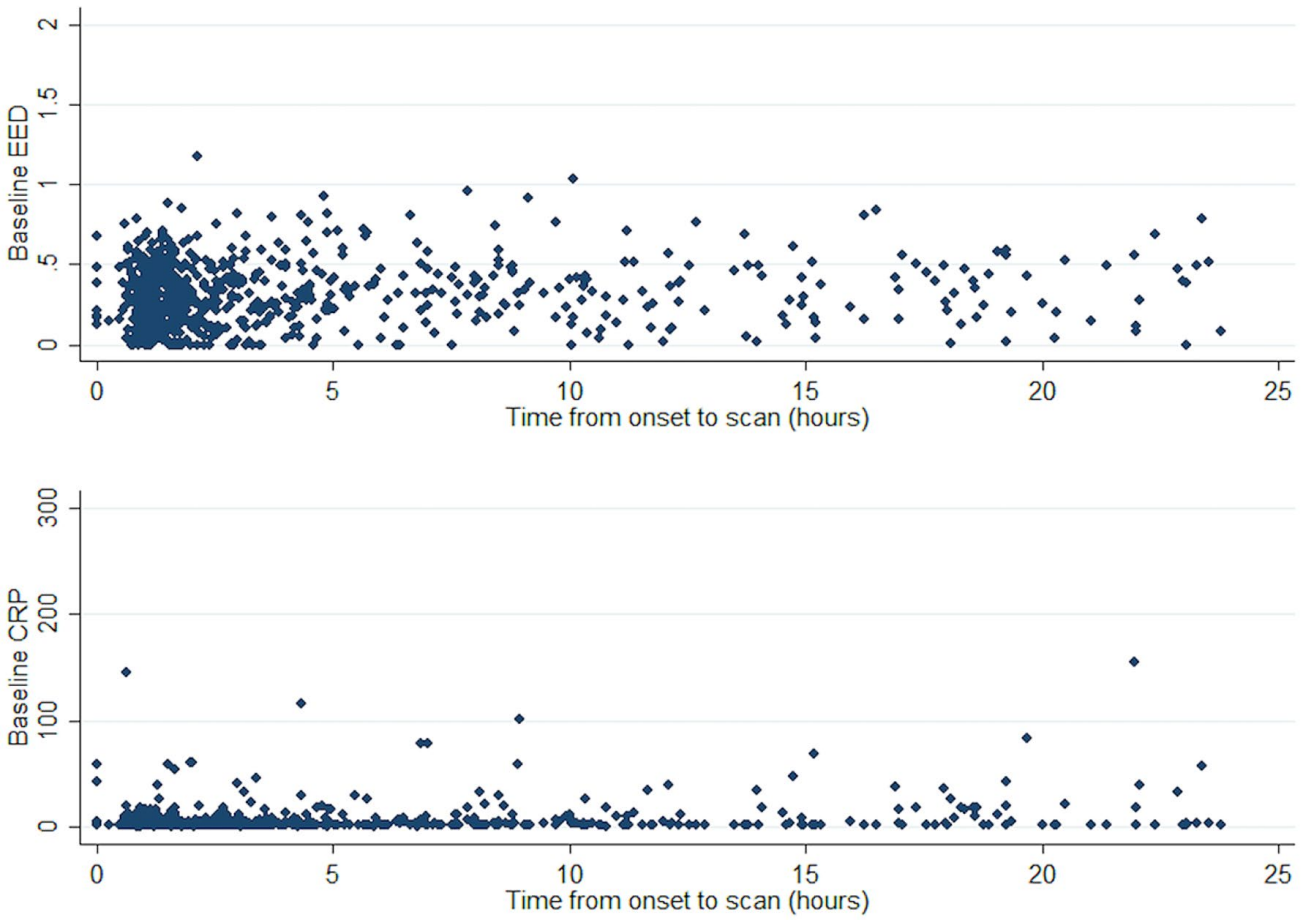
Course of CRP and EED against time from ICH onset. CRP, C-reactive protein; EED, Edema extension distance; and ICH, Intracerebral hemorrhage.

Both CRP and EED tended to be higher as time from onset increased (Figure 2). We thus conducted a sensitivity analysis in a subgroup of patients (*n* = 670) with available time to CRP measurement and time to CT scan from event as covariates. The association between baseline log-CRP and baseline EED was not statistically significant: for a 50% increase in baseline CRP baseline EED increased by 0.004 cm (95%CI < −0.002 to 0.009, *p* = 0.17).

### 3.3 Association between edema, CRP, and 30-day mortality

**Figure 3:**
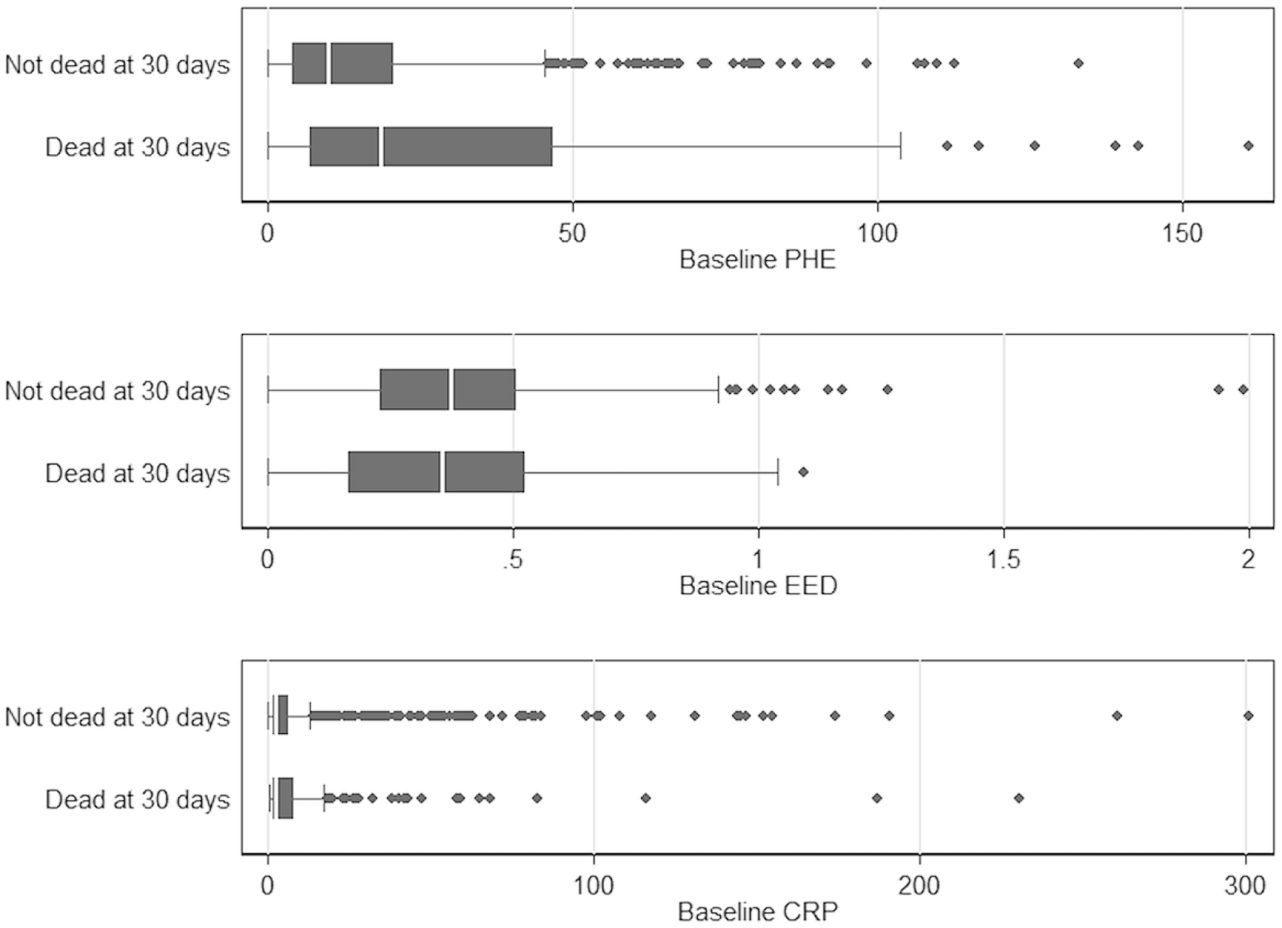
Boxplot comparing baseline PHE, EED, and CRP with mortality at 30 days, respectively. CRP, C-reactive protein; EED, Edema extension distance; and PHE, Perihematomal edema.

In initial unifactorial analyses, median (IQR) baseline PHE was larger in the patients who died within 30 days [23.7 (8.1–58.5) vs. 10.2 (3.8–10.2) mL, *p* < 0.001]. The median baseline CRP was lower in those who died by 30 days [3.0 (1.8–13.0) mg/L vs. 3.9 (2–10), *p* = 0.02] while there was no difference in median baseline EED [0.4 cm (0.2–0.6) vs. 0.4 (0.2–0.5), *p* = 0.78] in patients who died vs. the ones who survived (Figure 3).

Neither baseline log-CRP nor baseline log-PHE were independently associated with a hazard of 30-day mortality in the multifactorial Cox regression model (Table 3): HR 0.98 per 50% increase in CRP (95% CI 0.93–1.03, *p* = 0.41), HR 0.97 per 50% increase in PHE (95% CI 0.90–1.05, *p* = 0.51). In a sensitivity analysis, this result did not change when log-PHE volume was substituted by EED (keeping the model otherwise unchanged): EED was not significantly associated with mortality (HR 0.75, 95%CI 0.32–1.73, *p* = 0.50; full model results not shown).

**Table 3:** Multifactorial Cox regression model assessing the association between baseline C-reactive protein and baseline absolute edema volume and 30-day mortality.

	HR	95% CI	*p*-value
logPHE volume	0.94	0.78–1.13	0.511
logCRP	0.95	0.83–1.08	0.411
Age	1.03	1.01–1.04	<0.001
logICH volume	2.24	1.75–2.87	<0.001
IVH	1.64	1.16–2.30	0.005
Location			
Lobar	ref		-
Deep	1.97	1.37–2.83	<0.001
Brainstem	13.21	5.90–29.59	<0.001
Cerebellum	1.38	0.67–2.87	0.384
Statin use	1.26	0.87–1.82	0.227
GCS arrival, per point	0.85	0.81–0.88	<0.001
Baseline glucose, per what unit	1.02	0.99–1.06	0.257
Baseline systolic BP, per mmHg (suggest changing to 10 mmHg)	1.00	1.00–1.01	0.115
Cohort			
Helsinki	ref.	-	-
CROMIS	0.21	0.10–0.42	<0.001
Melbourne	0.96	0.52–1.87	0.965
Salford	1.54	0.96–2.48	0.074

BP, Blood pressure; CRP, C-reactive protein; GCS, Glasgow Coma Score; ICH, Intracerebral hemorrhage; IVH, Intraventricular hemorrhage; log, logarithm; and PHE, Perihematomal edema.

## 4 Discussion

In our large, multicenter ICH cohort we did not find an independent association between baseline CRP and baseline edema (measured by EED). Baseline edema (measured by PHE volume) in turn was not associated with 30-day mortality. None of the sensitivity analyses showed a significant association either.

Elevated CRP is commonly observed after ICH with median baseline CRP levels on admission of between 7 and 18 mg/L (10–12, 27, 28). Our median CRP was lower, at 2.5 mg/L. This could be due to earlier admission (shorter ictus to presentation time) and therefore earlier testing. It could as well be due to smaller ICH volumes although some studies have also reported smaller median ICH volumes compared to our cohort (22, 29–32). Natural history data of CRP kinetics beyond the first 24 h after ICH is limited. A previous group examined CRP kinetics in a cohort of 223 ICH patients and noted a median baseline CRP of 7.9 mg/L at a median of 93 min from ictus, which increased to 46 and 88.3 mg/L at 24 and 72 h, respectively (11), with an initial growth trajectory similar to that expected for PHE (5). In our cohort, higher baseline CRP was significantly associated with higher baseline EED in the unifactorial analysis but only showed a trend when adjusting for confounders (*p* < 0.001 and *p* = 0.055, respectively). The reduction in the association may be partly accounted for CRP and EED being independently associated with ICH volume. EED at later time points is hypothesized to reflect the intensity of the perihematomal inflammation relatively independently from the effect of baseline hematoma (25). CRP has been found in the perihematomal region from human subjects reflecting a zone of perihematomal inflammation after ICH (11). Although the inflammatory response to ICH in animal models begins within hours of onset, it increases in intensity over the next 48–72 h (33). Our study was restricted to patients within 24 h of symptom onset, thus considerably earlier than the maximal intensity of the inflammatory response. CRP takes up to 6 h to rise significantly after an acute stimulus (34), so measures within a few hours of symptom onset are likely to be more reflective of premorbid background inflammation than of the acute ICH. In our study, median time from onset to CRP measurement was 179 min (85–616 min), thus most measurements were earlier than any ICH-related rise would be expected to occur. The fact that we did not find a significant association most likely is due to the early measurement of CRP in the included cohort.

Higher baseline PHE volume was not independently associated with hazard of mortality within 30 days of ICH event. Absolute PHE volume and growth between 1 and 7 days after ICH ictus have been reported by various groups to be an independent poor prognostic marker after ICH (6, 7, 35–38). Recent studies using EED as an edema metric also demonstrated increasing edema growth at 72 h to be an independent and poor prognostic marker in ICH (5, 26).

We found no independent association between baseline CRP and survival. Several previous studies have investigated the significance of CRP and ICH outcome at different timepoints, with varied findings. In a study of 223 ICH patients admitted within 24 h of onset with daily CRP measurements over the first 72 h, the association between CRP at each time point, 30-day mortality and Glasgow Outcome Score was analyzed (11). In keeping with our findings, CRP on admission [median time from symptom onset to CRP 92 min (48–275 min)] was not associated with outcome. In their study, higher CRP at 24, 48, and 72 h was independently associated with a higher hazard of mortality and poor functional outcome. In a cohort of 961 ICH patients, higher CRP within 24 h of admission was found to be associated with unfavorable 3-month outcome (Glasgow Outcome Score 1–4) after adjusting for confounders (12). However, the exact timing of CRP measurements in relation to onset is not reported. These samples may thus be further from onset and thus less reflective of true baseline CRP. On the other hand, CRP measurement in our cohort could have been at a too early time point to catch the full extent of inflammation as we restricted it to patients within 24 h of symptom onset and the peak of intensity is reached after 48–72 h (33).

There are several limitations to this study. Firstly, our results focused on CRP and edema formation at baseline (<24 h) after ICH, so we do not have data on the evolution of CRP concentrations and edema beyond the first 24 h. Additionally, the exact timing of measurement of CRP was unknown in around a quarter of our study population. Although likelihood for relevant changes over the first few hours is low, evaluation in a further study with exact timing of blood values is needed to confirm this. Besides this, development over time beyond 24 h will also need to be evaluated prospectively in a systematic way with clear time-points of blood values taken as the effect is likely to be larger and show at a later time point. Secondly, although CRP is a sensitive and robust biomarker for systemic inflammation (18), we do not have complete data on CRP nor any data for other markers of systemic inflammatory response such as body temperature and peripheral leukocyte count. This again would be the scope of a prospective study with prespecified laboratory value collection and prespecified time-points. Also, there was a significant amount of missing data in several of the necessary variables including CRP (1,335) leading to the final inclusion of only 1,034 of the total 3,412 patients (30.3%), which probably limits the generalizability of this study. Thirdly, EED is a relatively new PHE metric increasingly being examined in PHE studies (5, 26, 39) after ICH and is a preferred edema metric to examine the perihematomal inflammatory response compared to absolute edema volume (25). The rationale behind this is that EED extends a consistent mean linear distance irrespective of ICH volume. Fourth, functional outcome data were not available in all the included studies on follow-up after ICH. The influence of the above findings on functional outcome should be the subject of future studies. Fifth, the included cohorts mostly only have baseline CT scans available as follow-up CT is not routinely performed in many centers, especially not following a specific time interval, which is normal for a standard clinical cohort. This again could be targeted in a future, prospective study. Lastly, patients recruited to the CROMIS study needed a consent given by themselves or a relative in order to be included into the study leading to exclusion of patients who died early during their hospitalization.

## 5 Conclusion

The early systemic inflammatory response, as measured by serum CRP, was not associated with baseline EED in our cohort most likely due to early measurement after symptom onset. This needs to be confirmed in independent, large prospective ICH cohort studies with predetermined inflammatory marker measurements at varying pre-specified time points to better understand the association between systemic inflammation and edema and show an effect if there really is one. Improving our understanding of the drivers of edema, how these change over time and the role of systemic inflammation will further inform future treatment approaches to improve outcomes after ICH.

## Data availability statement

The original contributions presented in the study are included in the article/supplementary material, further inquiries can be directed to the corresponding author.

## Ethics statement

The studies involving humans were approved by different ethical committees as multiple cohorts included. The studies were conducted in accordance with the local legislation and institutional requirements. The participants provided their written informed consent to participate in this study. Written informed consent was obtained from the individual(s) for the publication of any potentially identifiable images or data included in this article.

## Author contributions

OS: Conceptualization, Formal analysis, Investigation, Methodology, Writing – original draft, Writing – review & editing. IH: Conceptualization, Data curation, Formal analysis, Investigation, Methodology, Project administration, Validation, Visualization, Writing – original draft, Writing – review & editing. TW: Conceptualization, Data curation, Formal analysis, Investigation, Methodology, Writing – original draft, Writing – review & editing. CH: Formal analysis, Methodology, Validation, Visualization, Writing – original draft, Writing – review & editing. DWi: Conceptualization, Data curation, Formal analysis, Investigation, Writing – original draft, Writing – review & editing. DSh: Data curation, Formal analysis, Investigation, Methodology, Writing – original draft, Writing – review & editing. DSt: Data curation, Formal analysis, Investigation, Methodology, Writing – original draft, Writing – review & editing. JP: Data curation, Formal analysis, Investigation, Methodology, Writing – original draft, Writing – review & editing. TT: Data curation, Formal analysis, Investigation, Methodology, Writing – original draft, Writing – review & editing. AV: Data curation, Formal analysis, Investigation, Methodology, Writing – original draft, Writing – review & editing. GS: Data curation, Formal analysis, Investigation, Methodology, Writing – original draft, Writing – review & editing. SD: Data curation, Formal analysis, Investigation, Methodology, Writing – original draft, Writing – review & editing. DWe: Data curation, Formal analysis, Investigation, Methodology, Writing – original draft, Writing – review & editing. AM: Data curation, Formal analysis, Investigation, Methodology, Writing – original draft, Writing – review & editing. SA: Data curation, Formal analysis, Investigation, Methodology, Writing – original draft, Writing – review & editing. AP-J: Conceptualization, Data curation, Formal analysis, Investigation, Methodology, Project administration, Resources, Supervision, Validation, Visualization, Writing – original draft, Writing – review & editing.
